# (1*R*,2*S*,5*R*)-5-Methyl-2-[2-(4-nitro­phen­yl)propan-2-yl]cyclo­hexyl 2-(4-meth­oxy­phen­yl)-2,5-di­hydro-1*H*-pyrrole-1-carboxyl­ate: crystal structure and Hirshfeld analysis

**DOI:** 10.1107/S2056989018003092

**Published:** 2018-02-28

**Authors:** Julio Zukerman-Schpector, Monica Soto-Monsalve, Regina H. De Almeida Santos, Angelo H. L. Machado, Carlos Roque D. Correia, Mukesh M. Jotani, Edward R. T. Tiekink

**Affiliations:** a Laboratório de Cristalografia, Esterodinâmica e Modelagem Molecular, Departamento de Química, Universidade Federal de São Carlos, 13565-905 São Carlos, SP, Brazil; b Instituto de Química de São Carlos, Universidade de São Paulo, São Carlos, SP, Brazil; c Instituto de Química, Universidade Estadual de Campinas, UNICAMP, C.P. 6154, CEP. 13084-971, Campinas, São Paulo, Brazil; d Department of Physics, Bhavan’s Sheth R. A. College of Science, Ahmedabad, Gujarat 380001, India; e Centre for Crystalline Materials, School of Science and Technology, Sunway University, 47500 Bandar Sunway, Selangor Darul Ehsan, Malaysia

**Keywords:** crystal structure, pyrrolidine alkaloid, Heck reaction, Hirshfeld surface analysis

## Abstract

In the title compound, C_28_H_34_N_2_O_5_, the adjacent ester and nitro­benzene substituents are connected *via* an intra­molecular methyl­ene-C—H⋯π(nitrobenzene) inter­action and the mol­ecule approximates to a U-shape. The di­hydro­pyrrole ring (r.m.s. deviation = 0.003 Å) is almost co-planar with the carboxyl­ate residue [C_m_—N—C1—O_c_ (m = methine, c = carbox­yl) torsion angle = 1.8 (4)°] but is orthogonal to the 4-meth­oxy­benzene ring [dihedral angle = 84.34 (17)°]. In the crystal, methyl­ene-*C*—*H*⋯*O*(carbon­yl) inter­actions lead to linear supra­molecular chains along the *b*-axis direction, which pack without directional inter­actions between them. The analysis of the calculated Hirshfeld surface points to the importance of weak inter­atomic H⋯H, O⋯H/H⋯O and C⋯H/H⋯C contacts in the crystal.

## Abstract 2

The mol­ecule in the title compound approximates a U-shape with the nitro­benzene and ester substituents lying to the same side of the mol­ecule. In the crystal, linear supra­molecular chains are sustained by methyl­ene-C—H⋯O(carbon­yl) inter­actions.

## Chemical context   

The reaction of an unsaturated halide species with an alkene, in the presence of both a base and a organopalladium catalyst, to form a substituted alkene, is termed the Heck reaction (Heck, 1982 ▸; Crisp, 1998 ▸). As part of our investigations into the scope of the Heck reaction in the total, enanti­oselective and efficient synthesis of pyrrolidine alkaloids, such as the natural product (–)-codonopsinine (Severino & Correia, 2001 ▸), an enecarbamate containing the chiral auxiliary residue, 8-(4-nitro­phen­yl)menthol, was submitted to a Heck aryl­ation reaction with 4-meth­oxy­phenyl­diazo­nium tetra­fluoro­borate. The reaction yielded the title compound, 8-(4-nitro­phen­yl)menthyl 2-(4-meth­oxy­phen­yl)pyrroline-3-carboxyl­ate, (I), as the sole crystalline material (Machado, 2001 ▸). Herein, the crystal and mol­ecular structures of (I) are described along with an analysis of the calculated Hirshfeld surfaces.

## Structural commentary   

**Figure 1:**
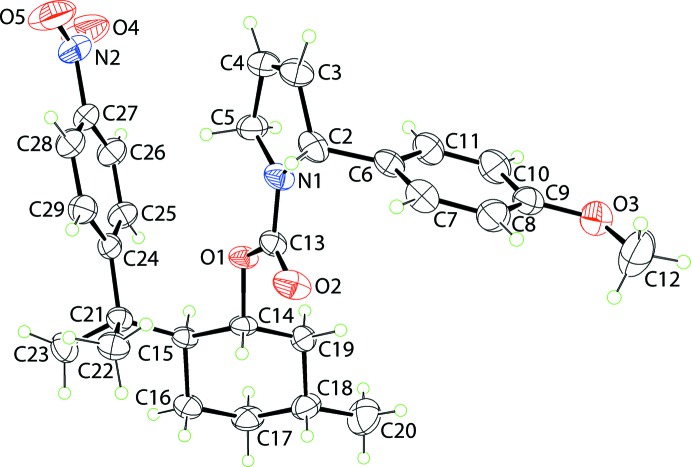
The mol­ecular structure of (I), showing the atom-labelling scheme and displacement ellipsoids at the 35% probability level.

The mol­ecular structure of (I), Fig. 1 ▸, comprises a 1-, 2- and 5-substituted cyclo­hexyl ring (chair conformation) with the chirality at these equatorially substituted centres, *i.e*. C14, C15 and C18, established from the synthesis, being *R*, *S* and *R*, respectively. The di­hydro­pyrrole ring is essentially planar, with an r.m.s. deviation of 0.003 Å for the five constituent atoms; the N1 and C5 atoms lie 0.037 (2) and 0.030 (3) Å to opposite sides of the plane. The chirality of the C2 centre is *R*. The carboxyl­ate residue is almost co-planar with the five-membered pyrrole ring as seen in the value of the C2—N1—C13—O2 torsion angle of 1.8 (4)°. However, the appended 4-meth­oxy­benzene ring is almost orthogonal to the pyrrole ring, forming a dihedral angle of 84.34 (17)°; the meth­oxy group is co-planar with the benzene ring with the C12—O3—C9—C10 torsion angle being 178.0 (4)°. In the same way, the nitro group is co-planar with the benzene ring to which it is connected with the O5—N2—C27—C28 torsion angle being 1.2 (5)°. In the mol­ecule, there is a close pyrrole-methyl­ene-C5—H⋯π(C24–C29) inter­action, Table 1 ▸, which connects the substituents at the cyclo­hexyl-C14 and C15 atoms which lie to the same side of the mol­ecule and which define a shape corresponding to the letter U.
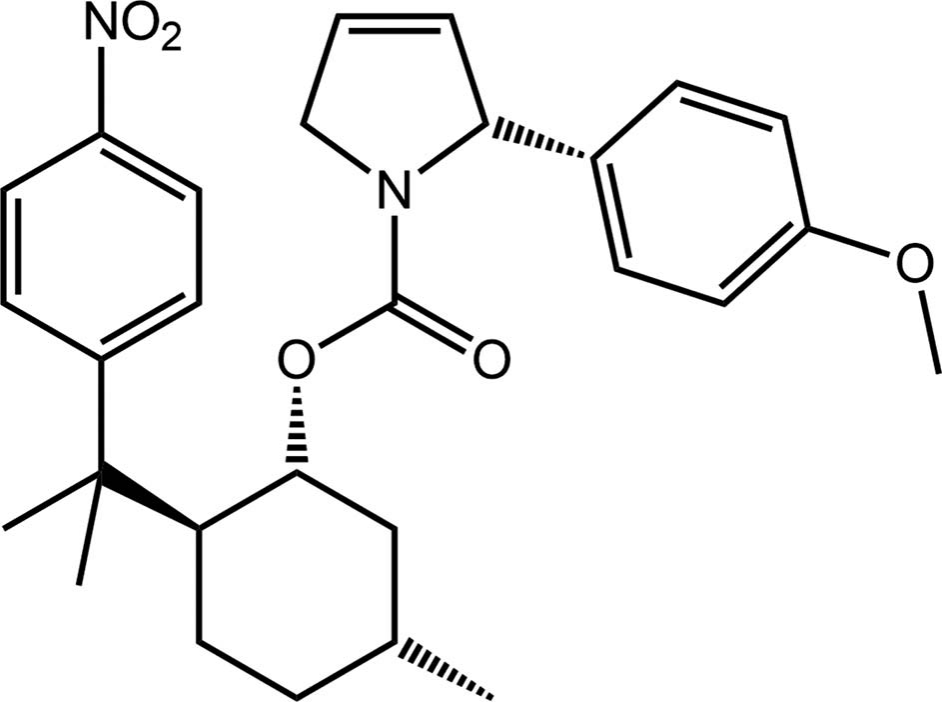


## Supra­molecular features   

**Figure 2:**
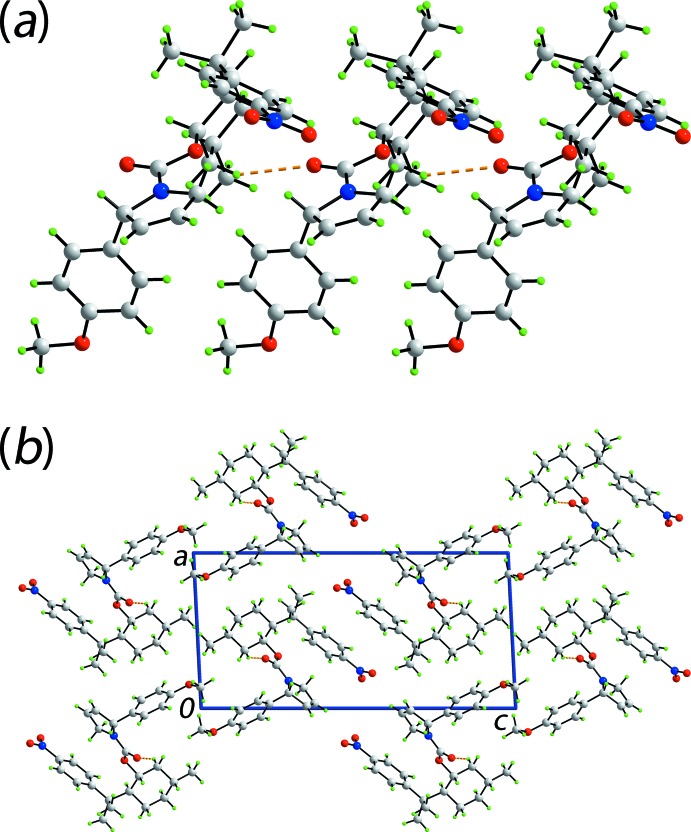
Mol­ecular packing in (I): (*a*) view of the supra­molecular chain along the *b* axis and (*b*) a view of the unit-cell contents shown in projection down the *b* axis. The C—H⋯O contacts are shown as orange dashed lines.

The mol­ecular packing of (I) features a number of weak non-covalent contacts as discussed below in the *Hirshfeld surface analysis* (§4). In accord with the distance criteria assumed in *PLATON* (Spek, 2009 ▸), there is only one directional inter­action of note, Table 1 ▸. Thus, methyl­ene-C19—H⋯O2(carbon­yl) inter­actions connect mol­ecules into a linear supra­molecular chain along the *b*-axis direction, Fig. 2 ▸ *a*. These assemble in the crystal with no directional inter­actions between them, Fig. 2 ▸ *b*.

## Hirshfeld surface analysis   

The Hirshfeld surfaces calculated for (I) were conducted as reported recently for a related organic mol­ecule (Zukerman-Schpector *et al.*, 2017 ▸) and provide information on the influence of short inter­atomic non-bonded contacts upon the mol­ecular packing.

**Figure 3:**
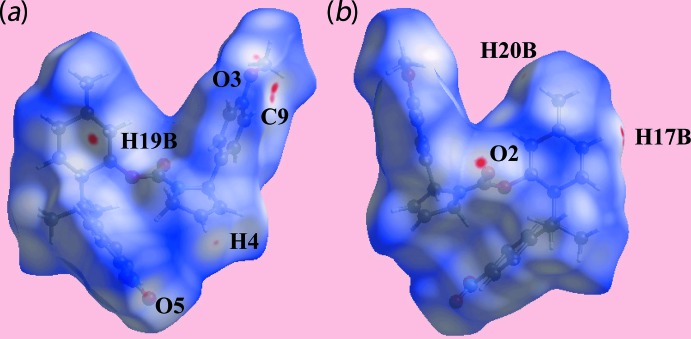
Two views of the Hirshfeld surface for (I) mapped over *d*_norm_ in the range −0.071 to +1.718 au.

**Figure 4:**
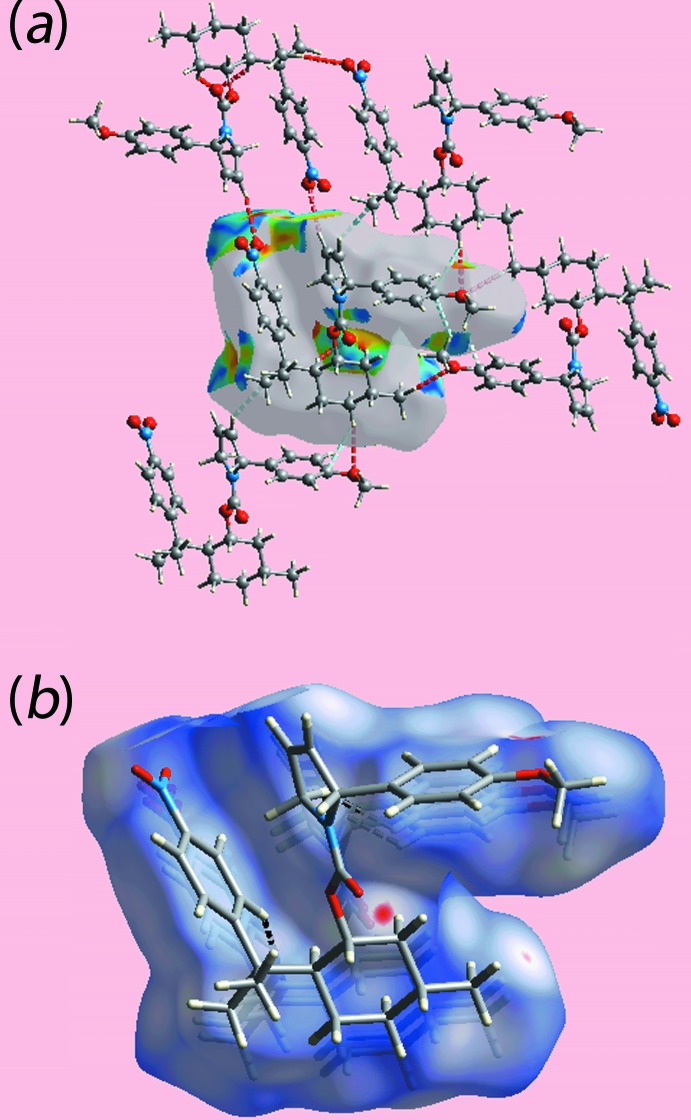
Views of Hirshfeld surfaces mapped (*a*) with shape-index property highlighting short inter­atomic O⋯H/H⋯O and C⋯H/H⋯C contacts by red and sky-blue dashed lines, respectively, and (*b*) over *d*_norm_ showing intra-layer inter­atomic H⋯H contacts by black dashed lines.

**Figure 5:**
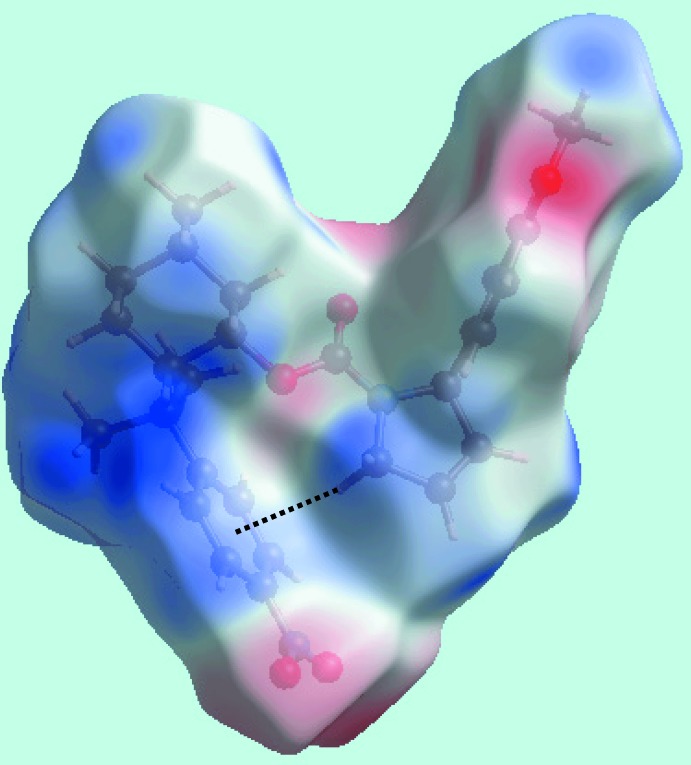
A view of the Hirshfeld surface mapped over the electrostatic potential for (I) in the range −0.079 to +0.038 au, highlighting the intra­molecular C—H⋯π contact by a black dotted line. The red and blue regions represent negative and positive electrostatic potentials, respectively.

With reference to Fig. 3 ▸, in addition to the bright-red spots near the methyl­ene-H19*B* and carbonyl-O2 atoms, representing the C—H⋯O inter­action listed in Table 1 ▸, the diminutive-red spots near the O3, C9 and H17*B* atoms, corresponding to short inter­atomic O3⋯H17*B* and C9⋯H17*B* contacts (Table 2 ▸), on the Hirshfeld surface mapped over *d*_norm_ suggest they also have some influence on the mol­ecular packing in the crystal. The effect of other short inter­atomic O⋯H/H⋯O and C⋯H/H⋯C contacts listed in Table 2 ▸ are also viewed as faint-red spots near the O3, H4, O5 and H20*B* atoms in Fig. 3 ▸. The influence of the short inter­atomic O⋯H, C⋯H and H⋯H contacts in the mol­ecular packing are also illustrated in Fig. 4 ▸ *a* and *b*, which show the Hirshfeld surface mapped over the shape-index property and *d*_norm_, respectively. The intra­molecular C—H⋯π contact between the pyrrole-H5*A* atom and the nitro­benzene ring [H5*A*⋯*Cg*(C24–C29) = 2.67 Å, C5⋯*Cg*(C24–C29) = 3.612 (3) Å and C—H5*A*⋯*Cg*(C24–C29) angle = 163°] is shown as a black-dotted line within the Hirshfeld surfaces mapped over the electrostatic potential in Fig. 5 ▸.

**Figure 6:**
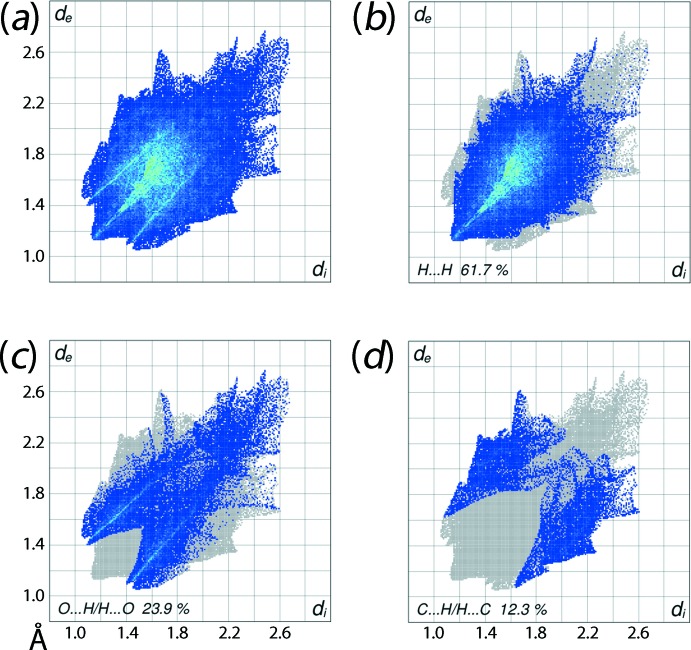
(*a*) The full two-dimensional fingerprint plot for (I) and fingerprint plots delineated into (*b*) H⋯H, (*c*) O⋯H/H⋯O and (*d*) C⋯H/H⋯C contacts.

The overall two-dimensional fingerprint plot for (I), Fig. 6 ▸ *a*, and those delineated into H⋯H, O⋯H/H⋯O and C⋯H/H⋯C contacts (McKinnon *et al.*, 2007 ▸) are illustrated in Fig. 6 ▸ *b*-*d*, respectively. The fingerprint plots also reflect the presence of the short inter­atomic contacts on the packing, Table 2 ▸. This is also evident from the percentage contribution from different inter­atomic contacts to the Hirshfeld surface summarized in Table 3 ▸: the H⋯H, O⋯H/H⋯O and C⋯H/H⋯C inter­atomic contacts make the greatest contribution to the Hirshfeld surface and account for 97.9% of the overall surface. The broad feather-like distribution of points with a peak at *d*_e_ + *d*_i_ ∼2.3 Å in the fingerprint plot delineated into H⋯H contacts in Fig. 5 ▸ *b* represent H⋯H contacts in the structure and make the greatest, *i.e.* 61.7%, contribution to the surface. The inter­atomic O⋯H/H⋯O contacts having a 23.9% contribution to the Hirshfeld surface arise from the C—H⋯O contact (Table 1 ▸) and short inter­atomic O⋯H/H⋯O contacts (Table 2 ▸), and are viewed as the pair of green aligned points beginning at *d*_e_ + *d*_i_ ∼2.6 Å and a pair of jaw-shaped distribution of points in the range *d*_e_ + *d*_i_ ∼2.5–2.6 Å in Fig. 6 ▸ *c*. The points distributed around the pair of forceps-like peaks at *d*_e_ + *d*_i_ ∼2.8 Å in the fingerprint plot delineated into C⋯H/H⋯C contacts (Fig. 6 ▸ *d*) represent the formation of such intra- and inter-layer contacts in the crystal. The small contribution from other inter­atomic contacts summarized in Table 3 ▸ appear to have a negligible impact on the mol­ecular packing.

## Database survey   

The (1*R*,2*S*,5*R*)-menthyl substrate is important as a chiral source for the synthesis of natural products and, as such, has been found in a number of crystal structures related to (I). Owing to the dictates of the chirality at the C1 and C2 positions, a parallel alignment of the substituents at these positions usually result in U-shaped geometries (Aoyagi *et al.*, 1998 ▸; Singh *et al.*, 1990 ▸; Streith *et al.*, 1995 ▸), except in circumstances where steric hindrance precludes such an arrangement (Comins & Killpack, 1992 ▸).

## Synthesis and crystallization   

As detailed previously (Machado, 2001 ▸), for the Heck aryl­ation of (1*R*,2*S*,5*R*)-5-methyl-2-[2-(4-nitro­phen­yl)propan-2-yl]cyclo­hexyl 2,3-di­hydro-1*H*-pyrrole-1-carboxyl­ate, a stoichiometric qu­antity of 4-meth­oxy­phenyl­diazo­nium tetra­fluoro­borate was used along with 1 mol equivalent of Pd^0^ and 400 mol equivalent of sodium acetate. The reaction was conducted in aceto­nitrile at room temperature for 15 min, yielding (1*R*,2*S*,5*R*)-5-methyl-2-[2-(4-nitro­phen­yl)propan-2-yl]cyclo­hexyl (2*S*)-2-(4-meth­oxy­phen­yl)-2,5-di­hydro-1*H*-pyrrole-1-carboxyl­ate and the title compound, (I), the latter being the only crystalline product, obtained as irregular colourless chunks by slow evaporation of an *n*-hexa­ne–ethyl acetate solution (8:2 *v*/*v*). M.p 378–380 K. ESI–MS (*m*/*z*) calculatedd for C_28_H_34_N_2_O_5_ [*M*]^+^ 478.24677, found 478.24676. [α]_D_^20^ = +85.6 9c = 0.7; ethyl­acetate). *R*_F_ = 0.40 (hexa­ne–ethyl acetate, 8:2 *v*/*v*).

The reported ^1^H and ^13^C NMR reflect the presence of two conformational rotamers in solution. ^1^H NMR (500 MHz, CCl_4_): δ [8.01 (*d*, *J* = 9 Hz) + 7.94 (*d*, *J* = 9 Hz) = 2H]; [7.43 (*d*, *J* = 9 Hz) + 7.16 (*d*, *J* = 9 Hz) = 2H]; [7.05 (*d*, *J* = 9 Hz) + 7.00 (*d*, *J* = 9 Hz) = 2H]; [6.77 (*d*, *J* = 9 Hz) + 6.70 (*d*, *J* = 9 Hz) = 2H]; 5.88 (*br d*, *J* = 6 Hz) + 5.67–5.59 (*m*) = 1H]; [5.67–5.59 (*m*) + 5.51 (*dd*, *J* = 7 Hz, 1 Hz) = 1H]; [5.27 (*br s*) + 5.19 (*br s*) = 1H]; 4.70 (*td*, *J* = 10 Hz and 5 Hz, 1H); [4.36 (*br d*, *J* = 15 Hz) + 4.21 (*m*) + 3.53 (*dd*, *J* = 15 and 5Hz) + 2.59 (*dd*, *J* = 15 and 2 Hz) = 2H]; [3.77 (*s*) + 3.72 (*s*) = 3H]; 2.04–0.49 (*m*, 11H); [1.43 (*s*) + 1.25 (*s*) = 3H]; [1.21 (*s*) + 1.11 (*s*) = 3H]. ^13^C NMR (75.5 MHz, CCl_4_): δ 159.1, 158.5, 151.6, 132.2, 130.9, 130.5, 128.0, 127.8, 125.9, 125.5, 123.9, 123.7, 122.3, 113.3, 113.1, 95.8, 73.5, 72.7, 67.3, 66.8, 54.6, 54.3, 51.7, 51.5, 42.4, 39.9, 34.3, 31.1, 30.2, 29.4, 26.0, 21.6, 21.5.

## Refinement details   

Crystal data, data collection and structure refinement details are summarized in Table 4 ▸. The C-bound H atoms were placed in calculated positions (C—H = 0.93–0.98 Å) and were included in the refinement in the riding model approximation, with *U*_iso_(H) set to 1.2–1.5*U*_eq_(C).

## Supplementary Material

Crystal structure: contains datablock(s) I, global. DOI: 10.1107/S2056989018003092/hb7734sup1.cif

Structure factors: contains datablock(s) I. DOI: 10.1107/S2056989018003092/hb7734Isup2.hkl

CCDC reference: 1825237

Additional supporting information:  crystallographic information; 3D view; checkCIF report

## Figures and Tables

**Table 1:** Hydrogen-bond geometry (Å, °) *Cg*1 is the ring centroid of the C24–C29 ring.

*D*—H⋯*A*	*D*—H	H⋯*A*	*D*⋯*A*	*D*—H⋯*A*
C5—H5*A*⋯*Cg*1	0.97	2.67	3.612 (3)	163
C19—H19*B*⋯O2^i^	0.97	2.60	3.472 (4)	150

Symmetry code: (i) 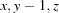.

**Table 2:** Summary of short inter­atomic contacts (Å) in (I)

Contact	Distance	Symmetry operation
H2⋯H5*B*	2.31	*x*, 1 + *y*, *z*
H7⋯H5*B*	2.28	*x*, 1 + *y*, *z*
H22*A*⋯H25	2.31	*x*, 1 + *y*, *z*
O3⋯H17*B*	2.52	1 + *x*, 1 + *y*, *z*
O3⋯H20*B*	2.56	2 − *x*, − 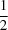 + *y*, 2 − *z*
O4⋯H4	2.56	2 − *x*, − 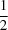 + *y*, 1 − *z*
O5⋯H22*C*	2.60	1 − *x*, − 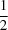 + *y*, 1 − *z*
C9⋯H17*B*	2.72	1 + *x*, 1 + *y*, *z*
C9⋯H12*C*	2.80	2 − *x*, − 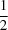 + *y*, 2 − *z*
C23⋯H3	2.84	−1 + *x*, −1 + *y*, *z*

**Table 3:** Percentage contributions of inter­atomic contacts to the Hirshfeld surface for (I)

Contact	Percentage contribution
H⋯H	61.7
O⋯H/H⋯O	23.9
C⋯H/H⋯C	12.3
N⋯H/H⋯N	1.1
O⋯O	0.7
C⋯O/O⋯C	0.2
C⋯C	0.1

**Table 4:** Experimental details

Crystal data	
Chemical formula	C_28_H_34_N_2_O_5_
*M*_r_	478.57
Crystal system, space group	Monoclinic, *P*2_1_
Temperature (K)	293
*a*, *b*, *c* (Å)	10.3142 (10), 6.1114 (8), 20.844 (3)
β (°)	92.83 (1)
*V* (Å^3^)	1312.3 (3)
*Z*	2
Radiation type	Mo *K*α
μ (mm^−1^)	0.08
Crystal size (mm)	0.40 × 0.25 × 0.20

Data collection	
Diffractometer	Enraf–Nonius TurboCAD4
Absorption correction	ψ scan (*CAD-4 EXPRESS*; Enraf–Nonius, 1989 ▸)
No. of measured, independent and observed [*I* > 2σ(*I*)] reflections	4246, 4145, 2310
*R*_int_	0.054
(sin θ/λ)_max_ (Å^−1^)	0.703

Refinement	
*R*[*F* ^2^ > 2σ(*F* ^2^)], *wR*(*F* ^2^), *S*	0.056, 0.144, 0.98
No. of reflections	4145
No. of parameters	320
No. of restraints	1
H-atom treatment	H-atom parameters constrained
Δρ_max_, Δρ_min_ (e Å^−3^)	0.27, −0.16
Absolute structure	No quotients, so Flack parameter determined by classical intensity fit
Absolute structure parameter	−1.1 (16)

Computer programs: *CAD-4 EXPRESS* (Enraf–Nonius, 1989 ▸), *XCAD4* (Harms & Wocadlo, 1995 ▸), *SIR2014* (Burla *et al.*, 2015 ▸), *SHELXL2014* (Sheldrick, 2015 ▸), *ORTEP-3 for Windows* (Farrugia, 2012 ▸), *DIAMOND* (Brandenburg, 2006 ▸), *MarvinSketch* (ChemAxon, 2010 ▸) and *publCIF* (Westrip, 2010 ▸).

## supplementary crystallographic information

### Crystal data

**Table:**

C_28_H_34_N_2_O_5_	*F*(000) = 512
*M**_r_* = 478.57	*D*_x_ = 1.211 Mg m^−^^3^
Monoclinic, *P*2_1_	Mo *K*α radiation, λ = 0.71073 Å
*a* = 10.3142 (10) Å	Cell parameters from 25 reflections
*b* = 6.1114 (8) Å	θ = 11.8–18.2°
*c* = 20.844 (3) Å	µ = 0.08 mm^−^^1^
β = 92.83 (1)°	*T* = 293 K
*V* = 1312.3 (3) Å^3^	Irregular, colourles
*Z* = 2	0.40 × 0.25 × 0.20 mm

### Data collection

**Table:**

Enraf–Nonius TurboCAD4 diffractometer	*R*_int_ = 0.054
Radiation source: Enraf–Nonius FR590	θ_max_ = 30.0°, θ_min_ = 2.3°
non–profiled ω/2θ scans	*h* = −14→14
Absorption correction: ψ scan (CAD-4 EXPRESS; Enraf–Nonius, 1989)	*k* = 0→8
	*l* = −29→0
4246 measured reflections	3 standard reflections every 60 min
4145 independent reflections	intensity decay: 1%
2310 reflections with *I* > 2σ(*I*)	

### Refinement

**Table:**

Refinement on *F*^2^	Hydrogen site location: inferred from neighbouring sites
Least-squares matrix: full	H-atom parameters constrained
*R*[*F*^2^ > 2σ(*F*^2^)] = 0.056	*w* = 1/[σ^2^(*F*_o_^2^) + (0.0751*P*)^2^] where *P* = (*F*_o_^2^ + 2*F*_c_^2^)/3
*wR*(*F*^2^) = 0.144	(Δ/σ)_max_ < 0.001
*S* = 0.98	Δρ_max_ = 0.27 e Å^−^^3^
4145 reflections	Δρ_min_ = −0.16 e Å^−^^3^
320 parameters	Absolute structure: No quotients, so Flack parameter determined by classical intensity fit
1 restraint	Absolute structure parameter: −1.1 (16)
Primary atom site location: structure-invariant direct methods	

### Special details

**Table:**

Geometry. All esds (except the esd in the dihedral angle between two l.s. planes) are estimated using the full covariance matrix. The cell esds are taken into account individually in the estimation of esds in distances, angles and torsion angles; correlations between esds in cell parameters are only used when they are defined by crystal symmetry. An approximate (isotropic) treatment of cell esds is used for estimating esds involving l.s. planes.

### Fractional atomic coordinates and isotropic or equivalent isotropic displacement parameters (Å^2^)

**Table:**

	*x*	*y*	*z*	*U*_iso_*/*U*_eq_	
N1	0.8239 (2)	0.1157 (4)	0.71804 (11)	0.0477 (6)	
N2	0.7587 (3)	−0.3619 (8)	0.48894 (14)	0.0797 (11)	
O1	0.66501 (17)	−0.1036 (3)	0.74757 (9)	0.0433 (5)	
O2	0.6807 (2)	0.2399 (4)	0.78790 (11)	0.0609 (6)	
O3	1.1526 (2)	0.4302 (5)	0.96516 (11)	0.0708 (7)	
O4	0.8084 (4)	−0.5387 (8)	0.48479 (16)	0.1195 (13)	
O5	0.7743 (3)	−0.2175 (7)	0.45058 (14)	0.1154 (13)	
C2	0.9005 (3)	0.3194 (5)	0.71908 (15)	0.0505 (8)	
H2	0.8431	0.4434	0.7086	0.061*	
C3	0.9859 (3)	0.2781 (7)	0.66428 (16)	0.0667 (10)	
H3	1.0448	0.3799	0.6498	0.080*	
C4	0.9688 (3)	0.0841 (8)	0.63905 (16)	0.0678 (11)	
H4	1.0140	0.0321	0.6047	0.081*	
C5	0.8683 (3)	−0.0446 (6)	0.67187 (15)	0.0553 (8)	
H5A	0.7983	−0.0899	0.6420	0.066*	
H5B	0.9055	−0.1725	0.6933	0.066*	
C6	0.9711 (3)	0.3593 (5)	0.78309 (15)	0.0475 (7)	
C7	0.9543 (3)	0.5462 (6)	0.81758 (16)	0.0564 (8)	
H7	0.9012	0.6560	0.7999	0.068*	
C8	1.0136 (3)	0.5782 (6)	0.87811 (16)	0.0605 (9)	
H8	1.0002	0.7076	0.9003	0.073*	
C9	1.0914 (3)	0.4197 (6)	0.90480 (15)	0.0546 (8)	
C10	1.1126 (3)	0.2279 (7)	0.87032 (17)	0.0638 (9)	
H10	1.1667	0.1196	0.8880	0.077*	
C11	1.0541 (3)	0.1994 (6)	0.81092 (16)	0.0574 (8)	
H11	1.0693	0.0715	0.7883	0.069*	
C12	1.1372 (5)	0.6248 (10)	1.0010 (2)	0.1029 (17)	
H12A	1.1692	0.7469	0.9775	0.154*	
H12B	1.1851	0.6128	1.0415	0.154*	
H12C	1.0469	0.6465	1.0082	0.154*	
C13	0.7197 (3)	0.0966 (5)	0.75437 (13)	0.0409 (6)	
C14	0.5631 (2)	−0.1508 (5)	0.79199 (12)	0.0399 (6)	
H14	0.5190	−0.0143	0.8024	0.048*	
C15	0.4655 (2)	−0.3067 (5)	0.75898 (12)	0.0386 (6)	
H15	0.5117	−0.4430	0.7505	0.046*	
C16	0.3628 (3)	−0.3597 (7)	0.80803 (14)	0.0563 (8)	
H16A	0.3163	−0.2271	0.8181	0.068*	
H16B	0.3006	−0.4633	0.7892	0.068*	
C17	0.4248 (3)	−0.4550 (6)	0.86912 (15)	0.0604 (9)	
H17A	0.4659	−0.5927	0.8592	0.072*	
H17B	0.3576	−0.4852	0.8989	0.072*	
C18	0.5250 (3)	−0.3043 (6)	0.90138 (13)	0.0568 (8)	
H18	0.4813	−0.1693	0.9135	0.068*	
C19	0.6253 (3)	−0.2468 (6)	0.85248 (13)	0.0474 (7)	
H19A	0.6868	−0.1424	0.8715	0.057*	
H19B	0.6729	−0.3777	0.8418	0.057*	
C20	0.5893 (4)	−0.4048 (9)	0.96163 (17)	0.0898 (14)	
H20A	0.5242	−0.4410	0.9912	0.135*	
H20B	0.6493	−0.3018	0.9813	0.135*	
H20C	0.6351	−0.5350	0.9504	0.135*	
C21	0.4069 (3)	−0.2208 (5)	0.69281 (13)	0.0416 (6)	
C22	0.3634 (3)	0.0191 (5)	0.69810 (16)	0.0530 (8)	
H22A	0.4380	0.1103	0.7068	0.079*	
H22B	0.3051	0.0330	0.7323	0.079*	
H22C	0.3201	0.0636	0.6584	0.079*	
C23	0.2859 (3)	−0.3568 (6)	0.67073 (15)	0.0565 (8)	
H23A	0.2172	−0.3298	0.6991	0.085*	
H23B	0.3075	−0.5096	0.6716	0.085*	
H23C	0.2582	−0.3151	0.6278	0.085*	
C24	0.5042 (2)	−0.2514 (5)	0.63983 (12)	0.0415 (6)	
C25	0.5699 (3)	−0.4489 (5)	0.63403 (14)	0.0511 (7)	
H25	0.5574	−0.5593	0.6638	0.061*	
C26	0.6534 (3)	−0.4855 (6)	0.58506 (14)	0.0573 (8)	
H26	0.6975	−0.6177	0.5822	0.069*	
C27	0.6697 (3)	−0.3229 (7)	0.54084 (13)	0.0557 (8)	
C28	0.6056 (3)	−0.1281 (7)	0.54376 (14)	0.0608 (9)	
H28	0.6167	−0.0207	0.5129	0.073*	
C29	0.5237 (3)	−0.0928 (6)	0.59349 (14)	0.0530 (8)	
H29	0.4806	0.0405	0.5959	0.064*	

### Atomic displacement parameters (Å^2^)

**Table:**

	*U*^11^	*U*^22^	*U*^33^	*U*^12^	*U*^13^	*U*^23^
N1	0.0418 (12)	0.0409 (14)	0.0610 (14)	−0.0140 (12)	0.0090 (11)	−0.0041 (13)
N2	0.078 (2)	0.115 (3)	0.0474 (16)	0.007 (2)	0.0169 (14)	−0.005 (2)
O1	0.0397 (10)	0.0395 (11)	0.0520 (10)	−0.0127 (9)	0.0150 (8)	−0.0037 (9)
O2	0.0527 (12)	0.0405 (12)	0.0911 (16)	−0.0101 (11)	0.0204 (11)	−0.0158 (13)
O3	0.0637 (13)	0.088 (2)	0.0603 (13)	−0.0025 (15)	−0.0009 (11)	0.0014 (14)
O4	0.141 (3)	0.129 (3)	0.094 (2)	0.037 (3)	0.059 (2)	−0.009 (2)
O5	0.131 (3)	0.142 (3)	0.0780 (19)	0.015 (3)	0.0538 (19)	0.027 (2)
C2	0.0430 (15)	0.0399 (17)	0.0684 (19)	−0.0163 (13)	−0.0002 (14)	0.0094 (14)
C3	0.0557 (19)	0.082 (3)	0.064 (2)	−0.034 (2)	0.0138 (16)	0.015 (2)
C4	0.0537 (18)	0.094 (3)	0.0569 (19)	−0.024 (2)	0.0169 (15)	0.004 (2)
C5	0.0540 (17)	0.060 (2)	0.0531 (16)	−0.0135 (17)	0.0160 (14)	−0.0087 (16)
C6	0.0368 (14)	0.0436 (17)	0.0625 (17)	−0.0125 (13)	0.0072 (12)	0.0034 (15)
C7	0.0477 (16)	0.051 (2)	0.070 (2)	−0.0005 (15)	0.0012 (15)	0.0039 (17)
C8	0.0575 (18)	0.059 (2)	0.0652 (19)	0.0002 (17)	0.0066 (15)	−0.0121 (18)
C9	0.0442 (15)	0.065 (2)	0.0552 (17)	−0.0055 (17)	0.0070 (13)	0.0046 (17)
C10	0.0536 (18)	0.060 (2)	0.077 (2)	0.0071 (18)	−0.0029 (17)	0.004 (2)
C11	0.0509 (16)	0.0486 (19)	0.072 (2)	−0.0037 (16)	0.0005 (15)	−0.0021 (17)
C12	0.127 (4)	0.110 (4)	0.070 (3)	0.007 (4)	−0.014 (3)	−0.028 (3)
C13	0.0362 (13)	0.0360 (15)	0.0506 (15)	−0.0067 (12)	0.0046 (11)	−0.0015 (13)
C14	0.0367 (13)	0.0397 (15)	0.0445 (14)	−0.0087 (12)	0.0159 (11)	−0.0044 (13)
C15	0.0331 (12)	0.0370 (15)	0.0466 (14)	−0.0065 (11)	0.0099 (10)	−0.0013 (12)
C16	0.0427 (15)	0.070 (2)	0.0573 (17)	−0.0166 (16)	0.0125 (13)	−0.0001 (18)
C17	0.0599 (18)	0.066 (2)	0.0576 (18)	−0.0162 (18)	0.0227 (15)	0.0081 (17)
C18	0.0626 (18)	0.064 (2)	0.0447 (15)	−0.0054 (17)	0.0128 (13)	0.0004 (16)
C19	0.0435 (14)	0.0507 (18)	0.0480 (15)	−0.0111 (14)	0.0032 (12)	−0.0050 (15)
C20	0.101 (3)	0.106 (4)	0.062 (2)	−0.016 (3)	0.002 (2)	0.021 (3)
C21	0.0383 (13)	0.0396 (16)	0.0470 (15)	−0.0021 (12)	0.0050 (12)	−0.0028 (13)
C22	0.0511 (17)	0.0450 (18)	0.0636 (18)	0.0069 (14)	0.0104 (15)	0.0012 (16)
C23	0.0448 (15)	0.060 (2)	0.0637 (18)	−0.0143 (15)	−0.0027 (13)	0.0000 (17)
C24	0.0393 (14)	0.0444 (16)	0.0406 (14)	−0.0068 (13)	−0.0005 (11)	−0.0010 (13)
C25	0.0631 (18)	0.0438 (17)	0.0472 (15)	−0.0021 (15)	0.0103 (14)	0.0015 (14)
C26	0.0671 (19)	0.0546 (19)	0.0504 (16)	0.0024 (17)	0.0063 (15)	−0.0097 (16)
C27	0.0545 (16)	0.078 (2)	0.0349 (14)	−0.0034 (18)	0.0029 (12)	−0.0059 (16)
C28	0.0618 (19)	0.075 (3)	0.0455 (16)	−0.0021 (19)	0.0035 (15)	0.0150 (18)
C29	0.0536 (16)	0.054 (2)	0.0516 (16)	0.0037 (16)	0.0034 (13)	0.0093 (16)

### Geometric parameters (Å, º)

**Table:**

N1—C13	1.350 (3)	C15—C16	1.543 (4)
N1—C5	1.463 (4)	C15—C21	1.569 (4)
N1—C2	1.474 (4)	C15—H15	0.9800
N2—O4	1.201 (5)	C16—C17	1.513 (4)
N2—O5	1.207 (5)	C16—H16A	0.9700
N2—C27	1.473 (4)	C16—H16B	0.9700
O1—C13	1.352 (3)	C17—C18	1.517 (5)
O1—C14	1.463 (3)	C17—H17A	0.9700
O2—C13	1.202 (3)	C17—H17B	0.9700
O3—C9	1.381 (4)	C18—C20	1.520 (5)
O3—C12	1.417 (6)	C18—C19	1.529 (4)
C2—C3	1.498 (5)	C18—H18	0.9800
C2—C6	1.508 (4)	C19—H19A	0.9700
C2—H2	0.9800	C19—H19B	0.9700
C3—C4	1.306 (6)	C20—H20A	0.9600
C3—H3	0.9300	C20—H20B	0.9600
C4—C5	1.494 (4)	C20—H20C	0.9600
C4—H4	0.9300	C21—C22	1.539 (4)
C5—H5A	0.9700	C21—C24	1.540 (4)
C5—H5B	0.9700	C21—C23	1.550 (4)
C6—C7	1.365 (5)	C22—H22A	0.9600
C6—C11	1.405 (4)	C22—H22B	0.9600
C7—C8	1.389 (4)	C22—H22C	0.9600
C7—H7	0.9300	C23—H23A	0.9600
C8—C9	1.359 (5)	C23—H23B	0.9600
C8—H8	0.9300	C23—H23C	0.9600
C9—C10	1.398 (5)	C24—C29	1.390 (4)
C10—C11	1.361 (5)	C24—C25	1.393 (4)
C10—H10	0.9300	C25—C26	1.386 (4)
C11—H11	0.9300	C25—H25	0.9300
C12—H12A	0.9600	C26—C27	1.371 (5)
C12—H12B	0.9600	C26—H26	0.9300
C12—H12C	0.9600	C27—C28	1.365 (6)
C14—C19	1.505 (4)	C28—C29	1.386 (4)
C14—C15	1.525 (4)	C28—H28	0.9300
C14—H14	0.9800	C29—H29	0.9300
			
C13—N1—C5	126.5 (2)	C17—C16—H16A	109.4
C13—N1—C2	120.4 (2)	C15—C16—H16A	109.4
C5—N1—C2	113.0 (2)	C17—C16—H16B	109.4
O4—N2—O5	122.6 (4)	C15—C16—H16B	109.4
O4—N2—C27	118.8 (4)	H16A—C16—H16B	108.0
O5—N2—C27	118.5 (4)	C16—C17—C18	113.0 (3)
C13—O1—C14	114.9 (2)	C16—C17—H17A	109.0
C9—O3—C12	117.4 (3)	C18—C17—H17A	109.0
N1—C2—C3	100.5 (3)	C16—C17—H17B	109.0
N1—C2—C6	112.6 (2)	C18—C17—H17B	109.0
C3—C2—C6	115.2 (2)	H17A—C17—H17B	107.8
N1—C2—H2	109.4	C17—C18—C20	112.4 (3)
C3—C2—H2	109.4	C17—C18—C19	108.2 (2)
C6—C2—H2	109.4	C20—C18—C19	111.2 (3)
C4—C3—C2	112.8 (3)	C17—C18—H18	108.3
C4—C3—H3	123.6	C20—C18—H18	108.3
C2—C3—H3	123.6	C19—C18—H18	108.3
C3—C4—C5	112.1 (3)	C14—C19—C18	111.9 (2)
C3—C4—H4	123.9	C14—C19—H19A	109.2
C5—C4—H4	123.9	C18—C19—H19A	109.2
N1—C5—C4	101.3 (3)	C14—C19—H19B	109.2
N1—C5—H5A	111.5	C18—C19—H19B	109.2
C4—C5—H5A	111.5	H19A—C19—H19B	107.9
N1—C5—H5B	111.5	C18—C20—H20A	109.5
C4—C5—H5B	111.5	C18—C20—H20B	109.5
H5A—C5—H5B	109.3	H20A—C20—H20B	109.5
C7—C6—C11	117.1 (3)	C18—C20—H20C	109.5
C7—C6—C2	122.2 (3)	H20A—C20—H20C	109.5
C11—C6—C2	120.7 (3)	H20B—C20—H20C	109.5
C6—C7—C8	122.3 (3)	C22—C21—C24	111.7 (2)
C6—C7—H7	118.8	C22—C21—C23	107.4 (3)
C8—C7—H7	118.8	C24—C21—C23	105.4 (2)
C9—C8—C7	119.8 (3)	C22—C21—C15	110.9 (3)
C9—C8—H8	120.1	C24—C21—C15	110.7 (2)
C7—C8—H8	120.1	C23—C21—C15	110.6 (2)
C8—C9—O3	125.0 (3)	C21—C22—H22A	109.5
C8—C9—C10	119.4 (3)	C21—C22—H22B	109.5
O3—C9—C10	115.6 (3)	H22A—C22—H22B	109.5
C11—C10—C9	120.1 (3)	C21—C22—H22C	109.5
C11—C10—H10	119.9	H22A—C22—H22C	109.5
C9—C10—H10	119.9	H22B—C22—H22C	109.5
C10—C11—C6	121.3 (3)	C21—C23—H23A	109.5
C10—C11—H11	119.3	C21—C23—H23B	109.5
C6—C11—H11	119.3	H23A—C23—H23B	109.5
O3—C12—H12A	109.5	C21—C23—H23C	109.5
O3—C12—H12B	109.5	H23A—C23—H23C	109.5
H12A—C12—H12B	109.5	H23B—C23—H23C	109.5
O3—C12—H12C	109.5	C29—C24—C25	117.2 (2)
H12A—C12—H12C	109.5	C29—C24—C21	122.4 (3)
H12B—C12—H12C	109.5	C25—C24—C21	120.3 (3)
O2—C13—N1	124.1 (3)	C26—C25—C24	121.8 (3)
O2—C13—O1	124.8 (2)	C26—C25—H25	119.1
N1—C13—O1	111.1 (2)	C24—C25—H25	119.1
O1—C14—C19	108.5 (2)	C27—C26—C25	118.6 (3)
O1—C14—C15	108.43 (19)	C27—C26—H26	120.7
C19—C14—C15	112.3 (2)	C25—C26—H26	120.7
O1—C14—H14	109.2	C28—C27—C26	121.8 (3)
C19—C14—H14	109.2	C28—C27—N2	119.7 (3)
C15—C14—H14	109.2	C26—C27—N2	118.5 (4)
C14—C15—C16	107.0 (2)	C27—C28—C29	118.9 (3)
C14—C15—C21	113.9 (2)	C27—C28—H28	120.5
C16—C15—C21	113.9 (2)	C29—C28—H28	120.5
C14—C15—H15	107.2	C28—C29—C24	121.7 (3)
C16—C15—H15	107.2	C28—C29—H29	119.2
C21—C15—H15	107.2	C24—C29—H29	119.2
C17—C16—C15	111.3 (2)		
			
C13—N1—C2—C3	−170.1 (3)	C19—C14—C15—C21	−175.3 (2)
C5—N1—C2—C3	6.0 (3)	C14—C15—C16—C17	−56.9 (4)
C13—N1—C2—C6	66.8 (3)	C21—C15—C16—C17	176.4 (3)
C5—N1—C2—C6	−117.1 (3)	C15—C16—C17—C18	58.3 (4)
N1—C2—C3—C4	−3.6 (4)	C16—C17—C18—C20	−178.3 (3)
C6—C2—C3—C4	117.7 (4)	C16—C17—C18—C19	−55.1 (4)
C2—C3—C4—C5	0.0 (5)	O1—C14—C19—C18	−179.1 (2)
C13—N1—C5—C4	169.8 (3)	C15—C14—C19—C18	−59.3 (3)
C2—N1—C5—C4	−6.0 (3)	C17—C18—C19—C14	55.0 (4)
C3—C4—C5—N1	3.6 (4)	C20—C18—C19—C14	179.0 (3)
N1—C2—C6—C7	−123.2 (3)	C14—C15—C21—C22	−46.9 (3)
C3—C2—C6—C7	122.4 (3)	C16—C15—C21—C22	76.2 (3)
N1—C2—C6—C11	54.4 (4)	C14—C15—C21—C24	77.7 (3)
C3—C2—C6—C11	−60.1 (4)	C16—C15—C21—C24	−159.3 (3)
C11—C6—C7—C8	−1.3 (4)	C14—C15—C21—C23	−165.9 (2)
C2—C6—C7—C8	176.4 (3)	C16—C15—C21—C23	−42.8 (3)
C6—C7—C8—C9	−0.1 (5)	C22—C21—C24—C29	−14.5 (4)
C7—C8—C9—O3	−178.1 (3)	C23—C21—C24—C29	101.8 (3)
C7—C8—C9—C10	1.2 (5)	C15—C21—C24—C29	−138.6 (3)
C12—O3—C9—C8	−2.7 (5)	C22—C21—C24—C25	170.2 (3)
C12—O3—C9—C10	178.0 (4)	C23—C21—C24—C25	−73.5 (3)
C8—C9—C10—C11	−0.9 (5)	C15—C21—C24—C25	46.1 (3)
O3—C9—C10—C11	178.4 (3)	C29—C24—C25—C26	1.3 (4)
C9—C10—C11—C6	−0.4 (5)	C21—C24—C25—C26	176.9 (3)
C7—C6—C11—C10	1.5 (4)	C24—C25—C26—C27	−0.9 (5)
C2—C6—C11—C10	−176.2 (3)	C25—C26—C27—C28	−0.3 (5)
C5—N1—C13—O2	−173.7 (3)	C25—C26—C27—N2	179.8 (3)
C2—N1—C13—O2	1.8 (4)	O4—N2—C27—C28	−175.6 (4)
C5—N1—C13—O1	5.8 (4)	O5—N2—C27—C28	1.2 (5)
C2—N1—C13—O1	−178.7 (2)	O4—N2—C27—C26	4.2 (5)
C14—O1—C13—O2	−8.9 (4)	O5—N2—C27—C26	−179.0 (4)
C14—O1—C13—N1	171.6 (2)	C26—C27—C28—C29	1.1 (5)
C13—O1—C14—C19	−88.2 (3)	N2—C27—C28—C29	−179.0 (3)
C13—O1—C14—C15	149.6 (2)	C27—C28—C29—C24	−0.7 (5)
O1—C14—C15—C16	177.8 (2)	C25—C24—C29—C28	−0.5 (4)
C19—C14—C15—C16	58.0 (3)	C21—C24—C29—C28	−175.9 (3)
O1—C14—C15—C21	−55.4 (3)		

### Hydrogen-bond geometry (Å, º)

*Cg*1 is the ring centroid of the C24–C29 ring.

**Table:**

*D*—H···*A*	*D*—H	H···*A*	*D*···*A*	*D*—H···*A*
C5—H5*A*···*Cg*1	0.97	2.67	3.612 (3)	163
C19—H19*B*···O2^i^	0.97	2.60	3.472 (4)	150

Symmetry code: (i) *x*, *y*−1, *z*.
